# Supervised discriminant analysis for droplet micro-magnetofluidics

**DOI:** 10.1007/s10404-015-1579-z

**Published:** 2015-04-10

**Authors:** Gungun Lin, Vladimir M. Fomin, Denys Makarov, Oliver G. Schmidt

**Affiliations:** Institute for Integrative Nanosciences, IFW Dresden, Helmholtzstr. 20, 01069 Dresden, Germany; Material Systems for Nanoelectronics, Chemnitz University of Technology, Reichenhainerstr. 70, 09107 Chemnitz, Germany

**Keywords:** GMR sensor, Supervised discriminant analysis, Droplet microfluidics, Ferrofluids

## Abstract

We apply the technique of supervised discriminant analysis (SDA) for in-flow detection in droplet-based magnetofluidics. Based on the SDA, we successfully discriminate *bivariant* droplets of different volumes containing different encapsulated magnetic content produced by a GMR-based lab-on-chip platform. We demonstrate that the accuracy of discrimination is superior when the correlation of variables for data training is included to the case when the spatial distribution of variables is considered. Droplets produced with differences in ferrofluid concentration of 2.5 mg/ml and volume of 200 pl have been identified with high accuracy (98 %), indicating the significance of SDA for e.g. the discrimination in magnetic immuno-agglutination assays. Furthermore, the results open the way for the development of a unique magnetofluidic platform for future applications in multiplexed droplet-based barcoding assays and screening.

**Electronic supplementary material:**

The online version of this article (doi:10.1007/s10404-015-1579-z) contains supplementary material, which is available to authorized users.

## Introduction

Micro-magnetofluidics has emerged and received growing interest owning to the extensive contribution of magnetism to microfluidics (Pamme 2007; Frenz et al. 2008; Wang 2008; Gijs et al. 2010; Nguyen 2012; Misuk et al. 2013). Adopting the format of optical flow cytometry, magnetic in-flow detection has demonstrated as first steps to determine the size and encapsulated magnetic content of droplets for droplet-based microfluidics (Pekas et al. 2004; Lin et al. 2013, 2014) and to enumerate magnetic beads (Shen et al. 2005; Loureiro et al. 2009; Mönch et al. 2011), magnetically labeled cells (Loureiro et al. 2011; Fernandes et al. 2014) and bacteria (Issadore et al. 2013) in a continuous flow. In addition, the analytical aspect of magnetic in-flow detection has been addressed based on the signal amplitude of magnetic sensors to differentiate diverse cells (Issadore et al. 2012) and encode various droplets (Lin et al. 2015) by employing magnetic nanoparticles of distinct magnetic moments and concentrations.

To fully explore the flow cytometric format in magnetofluidics, two parameters of the detected signals of droplets (signal amplitude and peak width) by magnetoresistive sensors have been used to access the concentration of magnetic nanoparticles and the size of droplets, respectively (Lin et al. 2013). By including more parameters for analysis, the performance of diagnostics and immunoassays can be boosted to a new level in terms of capacity and precision (Abraham et al. 2014). The key step to demonstrate the multiparametric analytical capability of a magnetofluidic platform is its feasibility to discriminate individual detection events into discrete populations, which lays the foundation for its further applications in analytical assays, clinical diagnostics and cell research. Nonetheless, this step has still not yet been addressed for magnetic in-flow detection. As a vital component of multiparametric analysis, supervised discriminant analysis (SDA) is a technique which is frequently used in computer science for machine learning and pattern recognition (McLachlan 2004) in financial system for risk estimation (Altman 1968) and in biomedical applications for diagnosis of human diseases (Machado et al. 2005; Veselkov et al. 2014). The excellent performance of SDA lies in its powerful discrimination ability of detection events based on reference databases and well-established algorithms. Therefore, it is of advantage to introduce SDA to magnetofluidics so that the potential of magnetic assays and diagnostics can be exploited to a new level.

Here, we apply for the first time the method of SDA to substantiate the discrimination of droplets encapsulating various magnetic content and dimensions. For this purpose, a magnetofluidic device based on integrated spin valve sensors has been fabricated. We study several crucial factors such as threshold values for parameter extraction as well as approaches for data training. By taking into account the correlation of the data with covariance matrix for the training, a first example of droplets produced with a concentration difference of 2.5 mg/ml and a volume difference of 200 pl can be successfully discriminated with high accuracy (~98 %), demonstrating its relevance for the discrimination of droplet changes for e.g. magnetic immuno-agglutination assays. It also paves the way for future development of a droplet-based magnetofluidic platform based on a magnetic encoding scheme for combinatorial analysis, highly multiplexed droplet assays or diagnostics.

## Experimental

### Fabrication of GMR-based magnetofluidic device

**Fig. 1:**
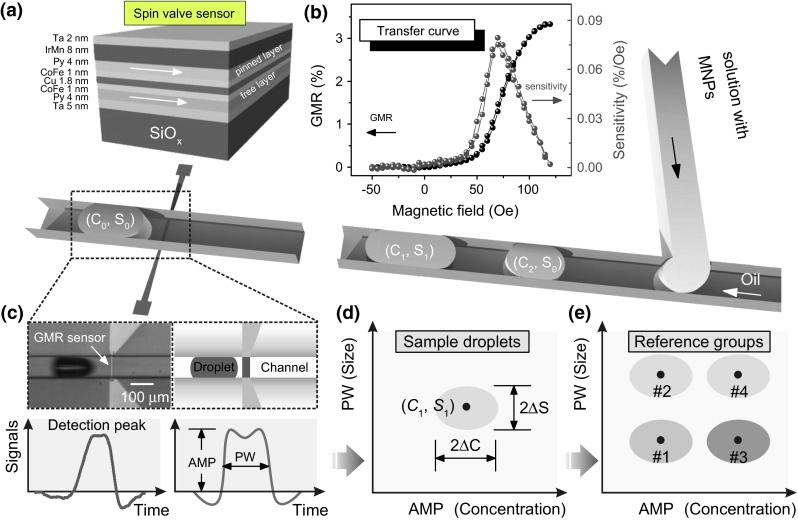
**a** Schematic sketch of the layer stack of spin valve sensors (*arrows* indicate the magnetization direction) as well as a lab-on-chip platform with integrated spin valve sensors for the detection of droplets of various sizes and encapsulated magnetic nanoparticles (MNPs). **b** Transfer curve of the integrated spin valve sensor (*black*) and its field-dependent sensitivity (*red*). **c** The optical micrograph (*top-left*) and schematic sketch of a droplet passing by a GMR sensor in a microfluidic channel (*top-right*). The real-time detection peak of a droplet (*bottom-left*) smoothened by adjacent averaging of 100 points and its schematic representation to extract the parameters for analysis (*bottom-right*). Here, the amplitude (AMP) and the peak width (PW) are used as parameters for multiparametric analysis. **d** Schematic multiparametric diagram for a group of sample droplets. The peak width (representing size of droplets) and the signal amplitude (representing concentration of encapsulated magnetic nanoparticles) are used as parameters. A droplet group can be labeled as (*C*_*i*_, *S*_*j*_). The average signal amplitude is *C*_*i*_ with a dispersion Δ*C*, the average peak width of the group of droplets is *S*_*j*_ with a dispersion Δ*S*. **e** Schematic multiparametric diagram summarizing multiple reference droplet groups produced with known properties which are labeled with # 1 to # 4 (color figure online)

A thermally oxidized silicon wafer with 600 nm oxides was chosen as the substrate. The wafer was spin coated with a photoresist (AZ 5214E, Microchems) at a spin speed of 4500 rpm, followed by baking at 90 °C for 4 min. Then, it was exposed with a mask aligner (Karl Suss, MJB4) for 2 s with a photomask with designed sensor structure. Afterward, the wafer was baked at 120 °C for 2 min and flood exposed for 30 s. Finally, the resist was developed by a developer (MIF 726, Microchems) to reveal the pattern of the designed sensor geometry. The designed pattern of the sensor has a rectangular shape and size of 6 µm × 100 µm. Afterward, a spin valve stack of: SiOx/Ta (5 nm)/Py (4 nm)/CoFe (1 nm)/Cu (1.8 nm)/CoFe (1 nm)/Py (4 nm)/IrMn (8 nm)/Ta (2 nm) (Fig. 1a), where Py = Ni_81_Fe_19_, was deposited onto the lithographically patterned substrate by magnetron sputtering. Before the sputter deposition, high vacuum condition with a base pressure around 9.5 × 10^−8^ mbar was achieved. During the deposition, Argon was used as the sputter gas. The pressure of Ar was 9.5 × 10^−4^ mbar, and the flow rate is kept constant at 10 sccm. An external magnetic field was applied during the deposition to induce exchange bias in the spin valve sensor. More details of the fabrication of the sensors are provided in ESI. After the deposition, a lift-off process was used to dissolve the photoresist. A second lithography step was followed to pattern electrical contacts. Ta (5 nm)/Cu (200 nm)/Ta (5 nm) were deposited by magnetron sputtering as the conducting materials.

The GMR curve of the integrated spin valve sensor (Fig. 1b; Fig. S1) shows that the sensor has a GMR ratio of 3.5 % and maximum sensitivity of about 0.08 %/Oe at a field of about 70 Oe. The sensor is pinned along the short axis of the stripe, and the free layer is used as the sensing layer. The sensitivity of the sensor is comparable to previous reported value (0.077 %/Oe) of a linear, hysteresis-free spin valve sensor used for magnetic in-flow detection (Pekas et al. 2004). However, for this study, the sensor is designed to have larger ferromagnetic coupling field (about five times larger than the reported value of about 15 Oe (Pekas et al. 2004; Melzer et al. 2012) to magnetize more magnetic nanoparticles, hence increasing the signal-to-noise ratio. The signal amplitude of the device in sensing 1 nl droplets encapsulating ferrofluids of 7.5 mg/ml is about 30 µV, which is six times larger than that of a previous demonstrated magnetofluidic device (about 5 µV) based on GMR multilayers (Lin et al. 2014).

A layer of polyetherimide (PEI) of thickness about 300 nm was spin coated onto the substrate and used as an insulation layer. Subsequently, SiO_2_ of 200 nm was deposited on top of the PEI insulation layer by e-beam evaporation, facilitating the bonding between polydimethylsiloxane (PDMS) channel and the substrate. The PDMS channel was fabricated by a mold–casting approach. To prepare the mold, the resist SU-8 50 was coated on a silicon wafer with a spin speed of 1000 rpm leading to a thickness of 100 µm. The SU-8 50 resist was baked at 60 °C for 5 min and at 90 °C for 15 min. Afterward, it was exposed by UV light to selective areas where the channel is and baked at 90 °C for 15 min. Finally, the resist was developed by a solvent (mr-Dev 600, micro resist technology GmbH). The fresh PDMS was prepared by mixing base polymers (SYLGARD 184 Silicone Elastomer KIT) with a curing agent in a weight ratio of 10:1. Degassing of PDMS in a vacuum chamber was carried out for 30 min to remove the bubbles. Afterward, the PDMS was poured into the SU-8 mold and cured at 180 °C for 5 min. The inlets of the channel were created by a biopsy puncher with a diameter of 1 mm. For the final assembly of the device, the PDMS channel and the chip were activated in an oxygen plasma (40 mw, O_2_ flow rate: 20 sccm) for 30 s. The channel and the substrate were brought into contact to achieve permanent bonding.

### Magnetoelectrical characterization and real-time detection

To characterize the sensor, the sample was placed in between pole shoes of an electromagnet. An uniform in-plane magnetic field was applied to the sensor, and the sensor resistance was obtained by 4-point measurement, i.e., a constant dc current was applied to the sensor, while the voltage change under a cycling magnetic field was recorded by a programmed multimeter (Keithley Model 2000). For the characterization, the magnetic field was swept in between ±300 Oe to saturate the sensors.

The experimental setup for the real-time detection of droplets (Fig. S2) is based on a Wheatstone bridge geometry to minimize the background noise level in order to achieve a high measurement sensitivity. An ac measuring current of 1 mA with a modulation frequency of 1 kHz was applied to the sensor. The differential voltage signal from the bridge was fed into a lock-in amplifier (SRS-830) to amplify the signal and reduce the noise. The analog output from the lock-in amplifier was picked up by an analog/digital (AD) converter (NI-USB 6009) with a sampling rate of 5 kHz and a measurement range of 2 mV. The ferrofluids (EMG 700 series, Ferrotec) used in the experiments are superparamagnetic nanoparticles (details are provided in the supplementary information), thus an external permanent magnet (AlNiCo 500, A1560, IBSMagnet) was used to magnetize the particles to induce net magnetic moments and to bias the sensor to the most sensitive region.

### Droplet formation

We used ferrofluid magnetic nanoparticles (EMG 700 series, Ferrotec) as the disperse phase and mineral oil (Sigma-Aldrich, M8410) with 5 % SPAN 80 as the continuous phase. Droplets encapsulating ferrofluids are formed at a T-junction. The total flow rate of oil and ferrofluids was kept at 15 nl/s with the flow speed about 1 mm/s, and only the ratio of the flow rates of the two phases was varied.

## Results and discussion

### Scheme of SDA with a droplet-based magnetofluidic platform

Figure 1 shows the scheme of SDA of emulsion droplets applied to a magnetofluidic device. Magnetic stray fields of a droplet passing by a GMR sensor are detected as voltage signals (Fig. 1b). The GMR sensor is a proximity sensor, and only local magnetic stray fields from the droplet are detected. Both simulations and experimental results (Pekas et al. 2004; Jeong et al. 2012; Lin et al. 2013) have shown that magnetic stray fields concentrate at two ends of the droplet as reflected by the local maximums and minimums near the rising and falling edges of the measured signals (Fig. 1c-bottom panel). The rising and falling edges of the magnetic fields correspond to the position of the two ends of the droplet, respectively. Therefore, the width of the detection peak can be correlated with the length of the droplet. In droplet microfluidics, droplets are produced under definite flow parameters that are characterized with a certain size and encapsulated magnetic content. By using the two parameters such as the peak width and the signal amplitude, it is feasible to group droplets of different sizes and concentrations of encapsulated magnetic content into different populations (Lin et al. 2013). For droplets produced with a certain set of flow parameters, the data are featured with a distribution of detection events of droplets by means of *C*_*i*_ (the average signal amplitude related to the concentration of magnetic nanoparticles), *S*_*j*_ (the average peak width related to the size of droplets) and respective dispersions (Δ*C*, Δ*S*) in a typical multiparametric diagram (Fig. 1d). The spread of detection events, analogous to conventional optical flow cytometry, comes from the variation in the size and encapsulated magnetic content of the generated droplets, flow conditions and environmental variations during the measurements. Thereby, for multiplexed analysis, prior reference measurements of multiple droplet groups should be produced by multiple sets of flow parameters which creates a series of data clouds (e.g., # 1 to # 4) spanning an entire diagram (Fig. 1e). The primary goal of SDA for a droplet-based magnetofluidic platform is to designate the sample droplets (Fig. 1d) into known reference groups (Fig. 1e).

### Extracting parameters

**Fig. 2:**
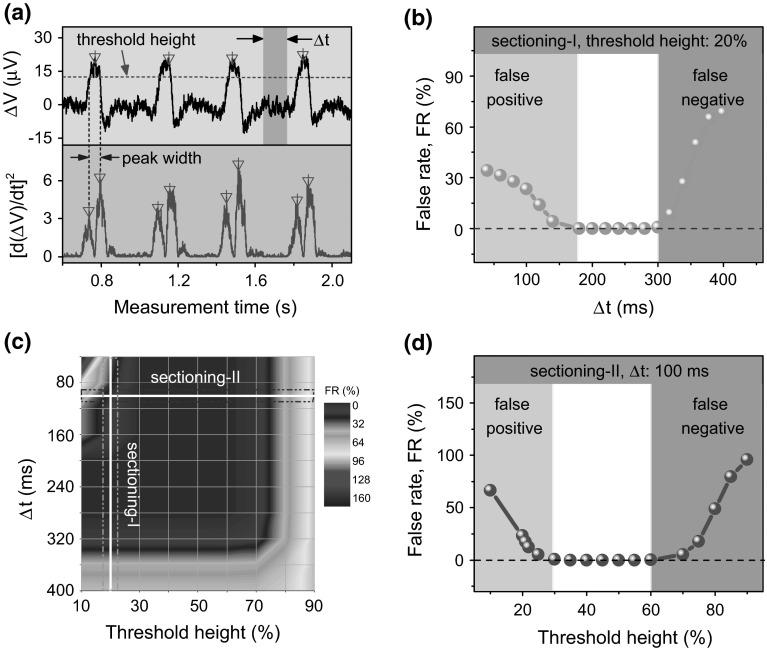
**a** Real-time signals of a train of droplets measured by the spin valve sensor (*top panel*). The sampling rate of the device is 5000 s^−1^.The squared first derivative of the voltage signals ((µV)^2^/s^2^) as a function of the measurement time. The *dashed line* and *colored rectangle* indicate the level of the threshold height and the local time interval (Δ*t*), respectively. The blank inverted triangles label the identified droplet detection peaks (*top*) as well as the local maxima of the squared first derivative of voltage signals (*bottom*) by the peak search algorithm. **b** The false rate (FR) of counting as a function of the local time interval (Δ*t*) with the threshold height of 20 %. **c** Projection of the 3D surface map of the false rate of counting with respect of Δ*t* and the value of threshold height. **d** The false rate as a function of the threshold height with a constant local time interval (Δ*t*) of 100 ms. Sectioning I and II are two line sectioning consistent with **b** and **d** (color figure online)

The signal amplitude is derived from the height of detection peaks, while the peak width is derived from the time difference between the rising and falling edges of the peak which can be obtained from the local maxima of the squared first derivative of signals (denoted by [d(Δ*V*)/d*t*]^2^, with Δ*V* the voltage signal and *t* the measurement time) (Fig. 2a). In all cases, it is crucial to effectively identify the detection peaks of droplets as well as the peak positions of the squared first derivative of signals. We applied an algorithm for the peak search which works in a way that a local maximum of the detection signal is searched for over a defined time interval (Δ*t*) by filtering the signals with a defined threshold height (in percentage of the maximum height of the total signals). Only a local signal maximum larger than the threshold height is identified as a peak found. Thus, various sets of the threshold height and Δ*t* may determine whether a group of droplets can be robustly extracted from a sample data.

We evaluate the efficiency of peak search by searching over a real-time data of a group of 500 droplets measured within a time frame of 180 s with different combinations of the threshold height (from 10 to 90 %) and Δ*t* (from 40 to 400 ms). The efficiency is evaluated according to the false rate (FR) of counting: FR = |(*N*_P_ − *N*_A_)|/*N*_A_, with *N*_P_ the number of droplets obtained from the search, *N*_A_ the actual number of droplets (here equals to 500). An example of the dependence of the FR on Δ*t* and the threshold height is shown in Fig. 2b and d, respectively. For a constant threshold height (20 %), the FR increases either with lower (<180 ms) or higher (>300 ms) Δ*t* (Fig. 2b). A similar trend is observed for the dependence of FR on the value of the threshold height with a constant Δ*t* of 100 ms (Fig. 2d). With less than 20 % or more than 60 % of the threshold height defined for the peak search, the FR also increases substantially. With a period of droplet detection (periodic droplets typical for droplet microfluidics) of ~300 ms (corresponding to a detection frequency of 3 droplets/s, as shown in Fig. 2a-top), it accounts for the increase in the FR if Δ*t* is set larger than 300 ms due to the fact that one of the neighboring peaks with the lower amplitude may be omitted during the peak search. Similarly, for the threshold height larger than 60 %, some peaks with amplitude lower than 60 % of the maximum height are not counted. Both lead to the negative false counting of droplets. For Δ*t* smaller than 180 ms and the value of the threshold height lower than 30 %, there is a positive false error owing to the fact that the signals of the same droplet peak can be counted more than once and the noise signals may be also taken into account. The boundary values of the threshold height indicate that within a droplet group, the lowest signal amplitude of a droplet and the highest noise level is about 60 and 30 % of the maximum amplitude of the signal, respectively.

The curves in Fig. 2b, d are in accordance with the two line sectionings of the projection of the 3D surface map of the FR (Fig. 2c). For a large set of the threshold height and Δ*t*, there exists a working band, where the droplet detection peaks can be robustly extracted without artifacts (FR = 0). This working band is located in the dark violet region of the surface map enclosed by these boundary values of the threshold height (30–60 %) and Δ*t* (180–300 ms). The same principle has been used also for the identification of the local maxima of the squared first derivative of signals in order to extract the peak width. The peak centers (labeled with blank triangles) can be robustly identified from the peak search algorithm as shown in Fig. 2a-bottom.

### Supervised discriminant analysis of droplets

**Fig. 3:**
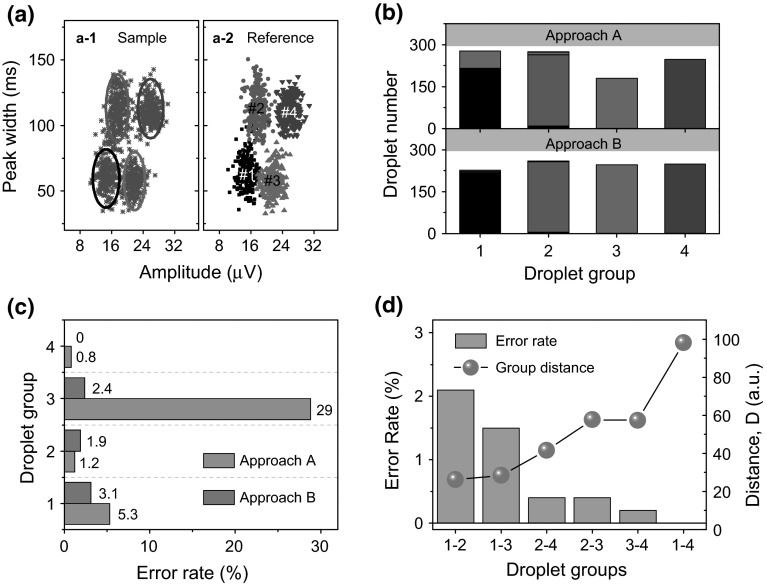
**a** Multiparametric density plot (*a-1*) for a large population of sample droplets with peak width and signal amplitude as parameters. *Ellipses* are guides to the eye. Multiparametric density plot (*a-2*) for detection events of droplets produced by the same flow parameters as used in (*a-1*) for droplet formation. Four different flow parameters are used to produce the four groups of droplets. Droplets belonging to the same reference group are labeled with the same color in (*a-2*). The concentration of ferrofluids and the average size of droplets for each group are 5.0 mg/ml and 200 pl for the group # 1, 5.0 mg/ml and 400 pl for the group # 2, 7.5 mg/ml and 200 pl for the group # 3, 7.5 mg/ml and 400 pl for the group # 4. **b** Predicted droplet populations of the large sample droplets in (*a-1*) which are allocated to the reference droplet groups labeled in (*a-2*) by SDA based on approach *A* (*top*) and *B* (*bottom*). The *bar* *height* indicates the population of droplets, and the *bar color* denotes which reference droplet group the allocated droplets originally belong to. **c** Comparison of the error rate of discrimination of droplets for each group based on approach *A* and *B*. **d** The error rate of SDA of every pair of selected droplet groups in **a** (*bar chart*). The *curve* (*solid spheres*) is the group distance between every two selected droplet groups (color figure online)

To perform this discrimination of droplets with SDA as pointed out in Fig. 1d, e, a bivariate sample data set comprising the two parameters (the signal amplitude and the peak width) has been obtained by setting the threshold values in the corresponding working band (Δ*t*: 200 ms, threshold height 40 %). A multiparametric density plot illustrating the data is shown in Fig. 3a-1. Each point in the plot represents a detection event of an emulsion droplet.

Although the large sample data show regional clustering of data points (indicated by ellipses in Fig. 3a-1), manual ‘gating’ or partitioning of the events through visual inspection into their own groups is subjective and difficult. A computational method of SDA can perform the task, which is to allocate an unknown population of droplets into known classified (reference) groups. As the droplets are produced with four definite flow parameters, reference droplet groups are those produced with the same four flow parameters (Fig. 3a-2). The basic principle of performing SDA is to first maximize the differences between groups with training data. However, regarding the training, the reference droplet group can be either fitting to a 2D Gauss function which only considers the distribution of data in a simpler case (approach A), or by evaluating its covariance matrix which summarizes the correlation in the variables (approach B). Both approaches have been used in this work to compare the quality of SDA (Details of the algorithm are given in the supplementary information). For approach *A*, a reference group *k* is fitting to a 2D Gauss function (G_*k*_) for the training, and the posterior probability ${q_{X_k}}$ of each sample droplet X belonging to a reference group *k* is calculated as:

$${q_{X_k}} = {{\text{G}}_k}(X)$$

Regarding approach *B*, Mahalanobis distance (Mahalanobis 1936) (${D_{X_k}}$) is used to measure the distance of a specified detection event *X* to a reference droplet group *k*. Together with the within-group covariance matrices Σ_*k*_, they can be further used to compute the posterior probability ${q_{X_k}}$ of detecting a droplet event (*X*) belonging to the *k* droplet group for the whole discrimination (Orglab 2014):

$$\log \left( {{q_{X_k}}} \right) = - \frac{1}{2}D_{X_k}^2 + \log \left( {\pi_k} \right) - \frac{1}{2}\log \left| {\varSigma_k} \right| + {c_0}\;$$

where *π*_*k*_ is the prior probability of detecting a droplet from the group *k* (*π*_*k*_ = 1/*n*_g_, with *n*_g_ the number of groups), and *c*_0_ is the normalizing constant. A droplet from the measured data set will thus be allocated to a droplet group which provides the highest posterior probability.

The result of SDA of the whole sample data with the two approaches is shown in Fig. 3b. The height of each bar indicates the predicted population for each group. The color denotes which reference droplet group the allocated droplets are originally belonging to. Compared with approach *B*, a larger proportion of droplets from group 3 (green) is misallocated to group # 1 (black) by approach A (Fig. 3b-top). This large misallocation is caused by the initial large spatial overlap of the reference groups # 1 and # 3 which cannot be differentiated with Gauss fitting. However, by taking into account the correlation of the variables (peak width and signal amplitude) with covariance matrix, the misallocation can be minimized. The error rates of discrimination of droplets with respect to each group based on the two approaches are summarized in Fig. 3c. For approach *A*, the total error rate is as high as 9 %. Despite of some overlap of data points in the training groups, approach B still shows an error rate below 4 % for all groups with a total error rate of only 2 %. It indicates that with approach B, for a droplet of a total volume of about 500 pl, the microfluidic device can successfully discriminate droplets produced with differences in the volume and concentration of encapsulated ferrofluids as small as 200 pl and 2.5 mg/ml. This represents the first demonstrated example of applying the method of SDA to analyze droplets of different properties for magnetofluidics, already enabling the device as a tool for the discrimination of droplet changes (in the order of a few nanoliters and mg/ml of magnetic particles) for magnetic immuno-agglutination assays, the analysis of which is by far still relied on optical measurements (Teste et al. 2013).

We further investigate the factors determining the successful rates of discrimination by performing SDA with approach B. Every two droplet groups in Fig. 3a were chosen for SDA, and the corresponding error rates of SDA were evaluated. The results are shown in Fig. 3d. The pairs of droplet groups are plotted in the order of increasing accuracy. The group distance between two droplet groups *D* is: *D*^2^ = *D*_*kl*_^2^ + *D*_*lk*_^2^, where *D*_*kl*_ is the Mahalanobis distance of the droplet group *k* to the mean value of group *l* and vice versa. The group distance defines the similarity of two droplet groups, which accounts for a reduced accuracy of discrimination with a smaller group distance. One the other hand, SDA of two droplet groups provides a mean to analyze the similarity of two flow parameters used for droplet formation. For instance, the error rate of discrimination with SDA on the two droplet groups, e.g., group # 1 and # 2 is about 2 % (Fig. 3d), which quantifies the similarity of the two droplet groups. The overlap between groups 1 and 3 results from the fact that the signal difference between the two groups is almost comparable to the noise level (3.5 µV). A larger error rate of SDA corresponds to a smaller group distance and a larger overlap. All the above suggests that flow conditions as well as the signal-to-noise ratio can be optimized to minimize the size dispersity and the similarity of droplet distribution so as to increase the screening capability and the success rate of SDA for multiplexed assays in practical applications. In such a case, the method of SDA can be used for the optimization of different flow parameters to produce well distinguishable droplet populations before they are put into use to encapsulate various biochemical species for screening.

## Conclusions

We highlight the technique of SDA to discriminate *bivariant* droplets of different sizes and encapsulated magnetic content. Two approaches with either Gauss function fitting or calculating covariance matrix have been compared to study the quality of analysis. The present GMR-based magnetofluidic device allows the discrimination of droplets produced with differences in the encapsulated magnetic content and volume as small as 2.5 mg/ml and 200 pl with high accuracy (98 %), already enabling the device as a tool relevant for the discrimination of droplet changes for magnetic immuno-agglutination assays. The method of SDA also provides a mean to evaluate different flow parameters used for droplet formation, which will be of great importance in droplet-based multiplexed assays so as to improve the screening capability. Different groups of droplets produced with various flow parameters should be optimized to be well distinguishable in order to achieve a successful prediction. Although the multiparametric analytical capability is demonstrated on droplet-based magnetic in-flow detection, we envision that this method of SDA could be extended for the analysis of synthesized magnetic beads or microgels used as substrates for suspension arrays. Magnetic microgels have been synthesized either by droplet microfluidics (Zhao et al. 2012) or batch preparation methods (Ménager et al. 2004). SDA in this case could be used to differentiate various magnetic bead substrates boosting the multiplexing capacity. We anticipate that the technique of multiparametric analysis for magnetic in-flow detection could provide perspectives to future development of highly multiplexed droplet-based assays or magnetic bead-based suspension arrays and screening.

## Electronic supplementary material

Supplementary material 1 (PDF 162 kb)

## References

[CR1] Abraham Y, Zhang X, Parker CN (2014). Multiparametric analysis of screening data: growing beyond the single dimension to infinity and beyond. J Biomol Screen.

[CR2] Altman EI (1968). Financial ratios, discriminant analysis and the prediction of corporate bankruptcy. J Finance.

[CR3] Fernandes A, Duarte C, Cardoso F (2014). Lab-on-chip cytometry based on magnetoresistive sensors for bacteria detection in milk. Sensors.

[CR4] Frenz L, El Harrak A, Pauly M (2008). Droplet-based microreactors for the synthesis of magnetic iron oxide nanoparticles. Angew Chem Int Ed Engl.

[CR5] Gijs MAM, Lacharme F, Lehmann U (2010). Microfluidic applications of magnetic particles for biological analysis and catalysis. Chem Rev.

[CR6] Issadore D, Chung J, Shao H (2012). Ultrasensitive clinical enumeration of rare cells ex vivo using a micro-hall detector. Sci Transl Med.

[CR7] Issadore D, Chung HJ, Chung J (2013). μHall chip for sensitive detection of bacteria. Adv Healthc Mater.

[CR8] Jeong I, Eu Y, Kim K, Hu X (2012). Magnetic sensor-based detection of picoliter volumes of magnetic nanoparticle droplets in a microfluidic chip. J Magn.

[CR9] Lin G, Baraban L, Han L (2013). Magnetoresistive emulsion analyzer. Sci Rep.

[CR10] Lin G, Makarov D, Melzer M (2014). A highly flexible and compact magnetoresistive analytic device. Lab Chip.

[CR11] Lin G, Makarov D, Medina-Sánchez M (2015). Magnetofluidic platform for multidimensional magnetic and optical barcoding of droplets. Lab Chip.

[CR12] Loureiro J, Ferreira R, Cardoso S (2009). Toward a magnetoresistive chip cytometer: integrated detection of magnetic beads flowing at cm/s velocities in microfluidic channels. Appl Phys Lett.

[CR13] Loureiro J, Andrade PZ, Cardoso S (2011). Magnetoresistive chip cytometer. Lab Chip.

[CR14] Machado RF, Laskowski D, Deffenderfer O (2005). Detection of lung cancer by sensor array analyses of exhaled breath. Am J Respir Crit Care Med.

[CR15] Mahalanobis P (1936). On the generalised distance in statistics. Proc Natl Inst Sci.

[CR16] McLachlan G (2004) Discriminant analysis and statistical pattern recognition. Wiley. ISBN:0471691151

[CR17] Melzer M, Lin G, Makarov D, Schmidt OG (2012). Stretchable spin valves on elastomer membranes by predetermined periodic fracture and random wrinkling. Adv Mater.

[CR18] Ménager C, Sandre O, Mangili J, Cabuil V (2004). Preparation and swelling of hydrophilic magnetic microgels. Polymer (Guildf).

[CR19] Misuk V, Mai A, Giannopoulos K (2013). Micro magnetofluidics: droplet manipulation of double emulsions based on paramagnetic ionic liquids. Lab Chip.

[CR20] Mönch I, Makarov D, Koseva R (2011). Rolled-up magnetic sensor: nanomembrane architecture for in-flow detection of magnetic objects. ACS Nano.

[CR21] Nguyen N-T (2012). Special issue on magnetic-based microfluidics. Microfluid Nanofluidics.

[CR22] Orglab (2014) http://www.originlab.com/doc/Origin-Help/DiscAnalysis-Algorithm

[CR23] Pamme N (2007). Continuous flow separations in microfluidic devices. Lab Chip.

[CR24] Pekas N, Porter MD, Tondra M (2004). Giant magnetoresistance monitoring of magnetic picodroplets in an integrated microfluidic system. Appl Phys Lett.

[CR25] Shen W, Liu X, Mazumdar D, Xiao G (2005). In situ detection of single micron-sized magnetic beads using magnetic tunnel junction sensors. Appl Phys Lett.

[CR26] Teste B, Ali-Cherif A, Viovy JL, Malaquin L (2013). A low cost and high throughput magnetic bead-based immuno-agglutination assay in confined droplets. Lab Chip.

[CR27] Veselkov KA, Mirnezami R, Strittmatter N (2014). Chemo-informatic strategy for imaging mass spectrometry-based hyperspectral profiling of lipid signatures in colorectal cancer. Proc Natl Acad Sci USA.

[CR28] Wang SX (2008). Advances in giant magnetoresistance biosensors with magnetic nanoparticle tags: review and outlook. IEEE Trans Magn.

[CR29] Zhao Y, Xie Z, Gu H (2012). Multifunctional photonic crystal barcodes from microfluidics. NPG Asia Mater.

