# Supplementary material for: Supervised discriminant analysis for droplet micro-magnetofluidics
Source: Microfluid Nanofluidics. 2015 Apr 10;19(2):457–64. doi: 10.1007/s10404-015-1579-z (PMC4562118; doi:10.1007/s10404-015-1579-z)
Supplement: Supplementary file 1 — Supplementary material 1 (PDF 162 kb) [file 10404_2015_1579_MOESM1_ESM.pdf]

Supplementary Information

Article Title:

Supervised discriminant analysis for droplet micro-magnetofluidics

Authors:

Gungun Lin,*[a,b] Vladimir M. Fomin,[a] Denys Makarov,[a] and Oliver G. Schmidt[a,b]

Addresses:

[a] Institute for Integrative Nanosciences, IFW Dresden, Helmholtzstr. 20, 01069 Dresden, Germany;

[b] Material Systems for Nanoelectronics, Chemnitz University of Technology, Reichenhainerstr. 70, 09107 Chemnitz, Germany

1. Magnetoelectrical characterization of spin valve sensor

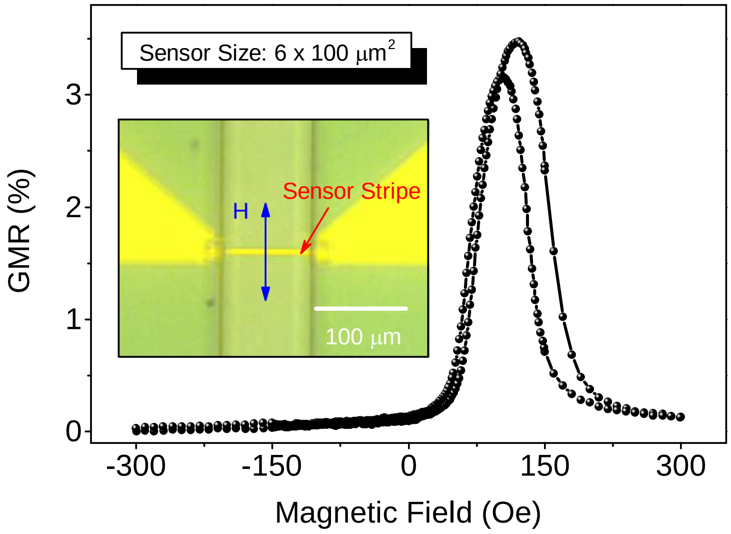

Fig. S1 Full range GMR curve of the spin valve sensor patterned into a size of 6 (width) x 100 (length) μm² (inset) which is integrated to the microfluidic channel. The pinning direction is along the short axis of the stripe.

2. Description of the algorithm of supervised discriminant analysis (SDA)

The principle of supervised discriminant analysis is to allocate unknown detection events into known classified groups. Prior training data (reference groups) should be used to maximize the differences between each reference group to which single detection events will be allocated. Two approaches have been used to compare the quality of analysis.

Approach A:

For the training of the data, the reference droplet group k is fitting to a 2D gauss function. A gauss function characterizing this group can be expressed as:

$$G_k = A \cdot \exp\left(-\left(\frac{(x-x_0)^2}{2\sigma_x^2} + \frac{(y-y_0)^2}{2\sigma_y^2}\right)\right)$$

here A is the amplitude; x, y is the parameters for the analysis, i.e. signal amplitude and peak width, respectively; x_0, y_0 is the center of gauss function; σ_x and σ_y is the x and y spreads of the function.

The posterior probability q_{X_k} of a droplet detection event X belonging to a reference group k is calculated by:

$$q_{X_k} = G_k(X)$$

Approach B:

The within-group covariance matrix of each group characterizes the group and is used to evaluate the Mahalanobis distance between each detection event within the group. the posterior probability of an unknown detection event from the measured sample can be evaluated based on the Mahalanobis distance of the detection event from each reference group and the covariance matrix of each reference group.

The within-group covariance matrix $\sum_k$ of a droplet group k is defined as [1]:

$$\Sigma_k = \begin{bmatrix} \Sigma_{11} & \Sigma_{12} \\ \Sigma_{21} & \Sigma_{22} \end{bmatrix} = \begin{bmatrix} E[(x_i-\overline{x_i})(x_i-\overline{x_i})] & E[(x_i-\overline{x_i})(x_j-\overline{x_j})] \\ E[(x_j-\overline{x_j})(x_i-\overline{x_i})] & E[(x_j-\overline{x_j})(x_j-\overline{x_j})] \end{bmatrix}$$

with Σ_{ij} (i, j = 1, 2) the covariance of the ith-variable x_i and jth-variables x_j, and $E(x_i, x_j)$ the expectation of the product of variables x_i and x_j, $\overline{x_i}$ and $\overline{x_j}$ being the mean values of the variable x_i and x_j in the droplet group, respectively.

For our present analysis, the variables x_i and x_j are the signal amplitude and the peak width of the detection events of droplets measured by a GMR sensor.

The Mahalanobis distance D_{X_k} is used to measure the distance of a detection event of a droplet $X=(x_i, x_j)$ from the distribution of the detection events in a droplet group k. It is given by [2]:

$$D_{X_k} = \sqrt{(X-\overline{X_k})^T \Sigma_k^{-1} (X-\overline{X_k})}$$

Here $\overline{X_k}$ is the mean value of the distribution of the droplet events in the droplet group k, Σ_k^{-1} is the inverse of the covariance matrix of the group k.

The posterior probability q_{X_k} of a detection event X belonging to a reference droplet group k can be obtained based on the covariance matrix Σ_k and the Mahalanobis distance D_{X_k} by the following expression:[3]

$$\log(q_{X_k}) = -\frac{1}{2} D_{X_k}^2 + \log(\pi_k) - \frac{1}{2}\log|\Sigma_k| + c_0$$

Here π_k is the prior probability of observing the droplet event in the droplet group k, c_0 is used to normalize the posterior probability of all detection events. As indicated, a larger distance of a detection event from the reference group and a broader spread of the detection events in the reference droplet group lead to a lower probability of observing the detection event in the corresponding reference group.

To perform the discrimination, the posterior probability of each droplet is computed for every reference group and those posterior probabilities are compared with each other to allocate the

droplet to the reference group which provides the largest posterior probability.

3. Description of real time in-flow measurements of droplets

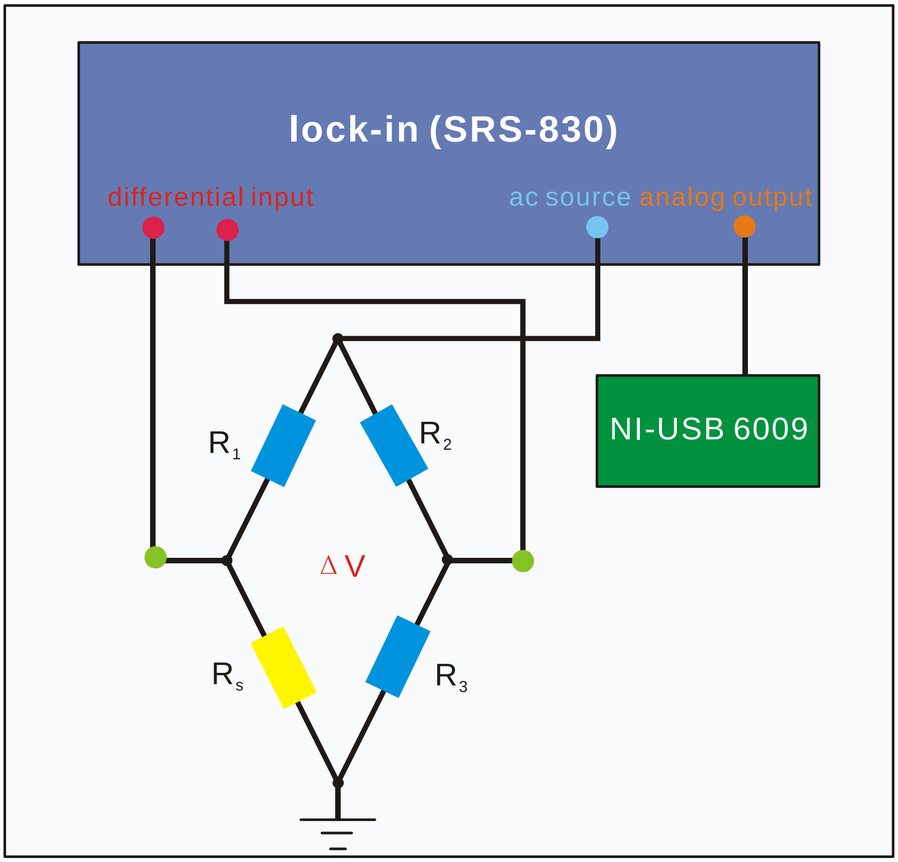

Fig. S2 Schematic of the electrical measurement setup for the real time detection of emulsion droplets in fluidics.

1. The measurement setup was established according to the Fig. S2. More specifically, the GMR sensor of the magnetofluidic device (R_s) is soldered with other trimmers R_1, R_2 and R_3 to form a Wheatstone bridge.
2. The bridge is powered with the ac voltage source from a lock-in (SRS 830) with a modulation frequency of 1 kHz.
3. The background voltage signal of the bridge is minimized by adjusting the resistance of the trimmers in order to enhance the measurement sensitivity.
4. After the background minimization, the differential voltage signal from the Wheatstone bridge is fed into the differential input of the lock-in, which is modulated with an internal reference signal of

the lock-in. With the modulation of the lock-in, the signal to noise ratio can be enhanced.

5. An external permanent magnet (AlNiCo 500, A1560, IBSMagnet) is placed below the sensor to bias the sensor and magnetize the ferrofluids. To correctly bias the sensor to the most sensitive region, the ferrofluid droplets are produced in the fluidic channel and passed across the sensor. The signal output from the droplets is monitored together with the careful adjustments of the position of the magnet until the maximum signal amplitude is achieved.
6. The analog output from the lock-in is sampled by a data acquisition box (NI USB 6009) and a computer program is made to control the settings of the real time measurements.

4. Protocol of the fabrication of GMR sensors

We describe two major steps to fabricate GMR sensors: (A) Substrate preparation (B) Magnetron sputtering deposition.

A. Substrate preparation

1. Choose thermally oxidized silicon wafers with 600 nm oxide (CrysTec GmbH) as the substrates. The oxide layer should be sufficiently thick to prevent any current shunting through the bottom substrate during electrical measurements.
2. Clean the wafers with acetone in an ultrasonic bath and afterwards with isopropanol to remove any contaminants on the surface to ensure the quality of subsequent photolithography process. Rinse the wafers with DI water and bake at 120 °C for 5 min to dehydrate to be ready for lithography.
3. Place the wafer on to a spin coater and apply the photoresist (AZ 5124E). Set the spin coating speed at 4500 rpm and spin the resist for 30s.
4. **Soft bake**: Transfer the photoresist-coated wafer to a hot plate at 90 °C for 4 min.
5. **Exposure**: Transfer the wafer to a mask aligner (MJB4, Karl Suss). Use a desired photomask with a designed sensor geometry (i.e. stripe). Expose the wafer for 2 s to initiate the photoactive compound.
6. **Post bake**: After exposure, place the wafer on a hot plate at 120 °C for 2 min. This step activates a cross-linking agent. Together with the exposed photoactive compound, the exposed area is insoluble in the developer.
7. Afterwards, expose the wafer without mask for 30 s. The previous unexposed area becomes soluble in the developer.
8. **Development**: Dip the wafer in a developer (MIF 726, Microchems) for 1 min to dissolve the

soluble area, which reveals a pattern of the photoresist with a designed geometry of the sensor (stripe). Rinse the wafer with DI water and dried with compressed air. A developer time should be well controlled to reveal the best resolution of final pattern.

B. Magnetron sputtering of GMR sensors

1. Transfer the lithographically patterned wafer into the deposition chamber of a magnetron sputtering machine. The sputtering machine should be installed with required targets (Ta, IrMn, CoFe, $Ni_{81}Fe_{19}$, Cu for spin valve sensors). The deposition rates (nm/s) are calibrated for each target prior to the deposition. Pump down the pressure of the chamber until a high vacuum condition is achieved. A base pressure of lower than 1×10^{-6} mbar should be reached prior to the deposition.
2. Until the base pressure is suitable for deposition, fill the chamber with Ar (as the sputter gas). The sputter pressure is about 9.4×10^{-4} mbar and a flow rate of 10 sccm which can be controlled by a flow and pressure controller. Presputter each target for 5 min to remove any contaminants on the target. When depositing each layer, the wafer is transferred under a specific target and a pressure-actuated shutter is used to control precisely the deposition time and the final layer thickness. The whole deposition process is of full automation which is remotely controlled via a computer program.
3. After the deposition, take out the sample from the chamber for lift-off.

Lift-off process

1. Immerse the wafer with deposited GMR sensors in the acetone (depending on the residual resist on the wafer, the whole lift-off time varies from 1 to several hours; optionally ultrasonic bath can be

used to assist the lift-off) to dissolve the photoresist to reveal the GMR sensors with a desired shape.

2. Rinse the wafer with isopropanol and dry it by compressed air.

Afterwards, a second lithography step is used to pattern electrical contacts for the GMR stack. This process is the same as described in **Step A**, except that to pattern the contacts, a different photomask with a designed geometry of electrical contacts is used. The pattern of contacts should be precisely aligned with the previous deposited GMR stack before exposure with the mask aligner. When a second lithography step is finished, the sample is again brought to the magnetron sputtering machine to deposite the contacts. In this case, Ta (5 nm) /Cu (200 nm) /Ta (5 nm) is used as the material.

4. Datasheet of ferrofluid magnetic nanoparticles

The details of the ferrofluid particles (EMG 700 series, ferrotec) are reproduced from [4]:

Manufacturer name: Ferrotec (USA) Corporation

Carrier Liquid	Water	
Nominal Particle Diameter	10 nm	
	CGS Units	SI Units
Saturation Magnetization (Ms)	325 Gauss	32.5 mT
Viscosity @27°C	<5 cP	<5 mPa·s
Density @25°C	1.29 g/cc	1.29 x 10^3kg/m^3
Initial Magnetic Susceptibility	1	12.57
Magnetic Particle Concentration	5.8 % vol.	
pH	~7	
Nature of Surfactant	Anionic	

References

1. N. H. Timm, *Applied Multivariate Analysis*, Springer Science & Business Media, 2002.
2. R. De Maesschalck, D. Jouan-Rimbaud, and D. L. Massart, *Chemom. Intell. Lab. Syst.*, 2000, **50**, 1–18.
3. http://www.originlab.com/www/helponline/Origin/en/UserGuide/Algorithm_(Discriminant_Analysis).html
4. https://www.ferrotec.com/index.php?id=audioFluid&vfp_id=110
